# Comparative transcriptome and weighted correlation network analyses reveal candidate genes involved in chlorogenic acid biosynthesis in sweet potato

**DOI:** 10.1038/s41598-022-06794-4

**Published:** 2022-02-17

**Authors:** Jing Xu, Jiahong Zhu, Yanhui Lin, Honglin Zhu, Liqiong Tang, Xinhua Wang, Xiaoning Wang

**Affiliations:** 1 Institute of Cereal Crops, Hainan Academy of Agricultural Sciences, Key Laboratory of Crop Genetics and Breeding of Hainan Province, Haikou, 571100 China; 2 Institute of Tropical Biosciences and Biotechnology, Chinese Academy of Tropical Agricultural Sciences, Haikou, 571101 China; 3 Sanya Research Institute of Hainan Academy of Agricultural Sciences, Sanya, 572000 China

**Keywords:** Biochemistry, Genetics, Plant sciences

## Abstract

Chlorogenic acids (CGAs) are important secondary metabolites produced in sweet potato. However, the mechanisms of their biosynthesis and regulation remain unclear. To identify potential genes involved in CGA biosynthesis, analysis of the dynamic changes in CGA components and RNA sequencing were performed on young leaves (YL), mature leaves (ML), young stems (YS), mature stems (MS) and storage roots (SR). Accordingly, we found that the accumulation of six CGA components varied among the different tissues and developmental stages, with YS and YL recording the highest levels, while SR exhibited low levels. Moreover, the transcriptome analysis yielded 59,287 unigenes, 3,767 of which were related to secondary-metabolite pathways. The differentially expressed genes (DEGs) were identified based on CGA content levels by comparing the different samples, including ML vs. YL, MS vs. YS, SR vs. YL and SR vs. YS. A total of 501 common DEGs were identified, and these were mainly implicated in the secondary metabolites biosynthesis. Additionally, eight co-expressed gene modules were identified following weighted gene co-expression network analysis, while genes in darkgrey module were highly associated with CGA accumulation. Darkgrey module analysis revealed that 12 unigenes encoding crucial enzymes (PAL, 4CL, C4H, C3H and HCT/HQT) and 42 unigenes encoding transcription factors (MYB, bHLH, WD40, WRKY, ERF, MADS, GARS, bZIP and zinc finger protein) had similar expression patterns with change trends of CGAs, suggesting their potential roles in CGA metabolism. Our findings provide new insights into the biosynthesis and regulatory mechanisms of CGA pathway, and will inform future efforts to build a genetically improve sweet potato through the breeding of high CGA content varieties.

## Introduction

Chlorogenic acids (CGAs) are phenolic compounds widely found in plants with a broad spectrum of biological activities, such as anti-inflammatory, antioxidant activity, antibacterial, anti-obesity, antiviral, antidiabetic, anti-microbial, hypolipidemic and anti-hypertension^1–3^. Three biosynthetic routes have been proposed for CGA production in plants^4^. The first pathway involves the caffeic acid coenzyme A and quinic acid catalysis by hydroxycinnamoyl-CoA quinate hydroxycinnamoyl transferase (HQT) to generate CGA^5^. In contrast, the second route entails generating CQAs with caffeoyl glucoside as an activated intermediate catalyzed by hydroxyl cinnamoyl D-glucose:quinate hydroxycinnamoyl transferase (HCGQT)^6^. Third, CGA is biosynthesised from *p*-coumaroyl quinic acid and catalyzed by hydroxyl cinnamoyl CoA:shikimate/quinate hydroxycinnamoyl transferase (HCT) and *p*-coumarate 3′-hydroxylase (C3H)^7^. Notably, both HQT and HCT enzymes are members of plant-specific acyl-CoA-dependent acyltransferases BAHD superfamily^8,9^. Among the three distinct pathways, the HQT-mediated pathway is considered the principal route for CGA biosynthesis^1,9^. Moreover, various studies have confirmed that overexpression or suppression of *HQT* in many plants leads to significant changes in CGA levels in most plants^1,10–15^. However, the specific mechanisms involved in CGA biosynthesis regulation in plants are still not well understood.

Sweet potato [*Ipomoea batatas* (L.) Lam.] is one of the most important crops globally mainly utilized as food, feed and industrial raw material^16^. Several studies have reported that sweet potatoes contain many phenolic compounds, such as CGAs. These compounds have multiple physiological and health functions, including confering the resistance against pests and diseases^17–20^. CGA accumulation varies in different tissues and development stages of sweet potatoes. Therefore, it is critical to understand CGA biosynthesis patterns during development and their regulation at the transcriptional level. However, the underlying molecular basis and regulation of CGA biosynthesis in sweet potato remain unclear^21^. In the present study, sweet potato CGA composition and content were investigated in different tissues and development stages, including young leaves (YL), mature leaves (ML), young stems (YS), mature stems (MS) and storage roots (SR). Additionally, RNA sequencing, differentially expressed gene (DEG) analysis and weighted gene co-expression correlation network analysis (WGCNA) were performed to examine and identify CGA biosynthesis-associated genes. This study provides valuable information for future research on understanding the mechanism of CGA metabolism pathway in sweet potatoes.

## Results

### Chlorogenic acid content and composition analysis in sweet potato tissues

**Figure 1:**
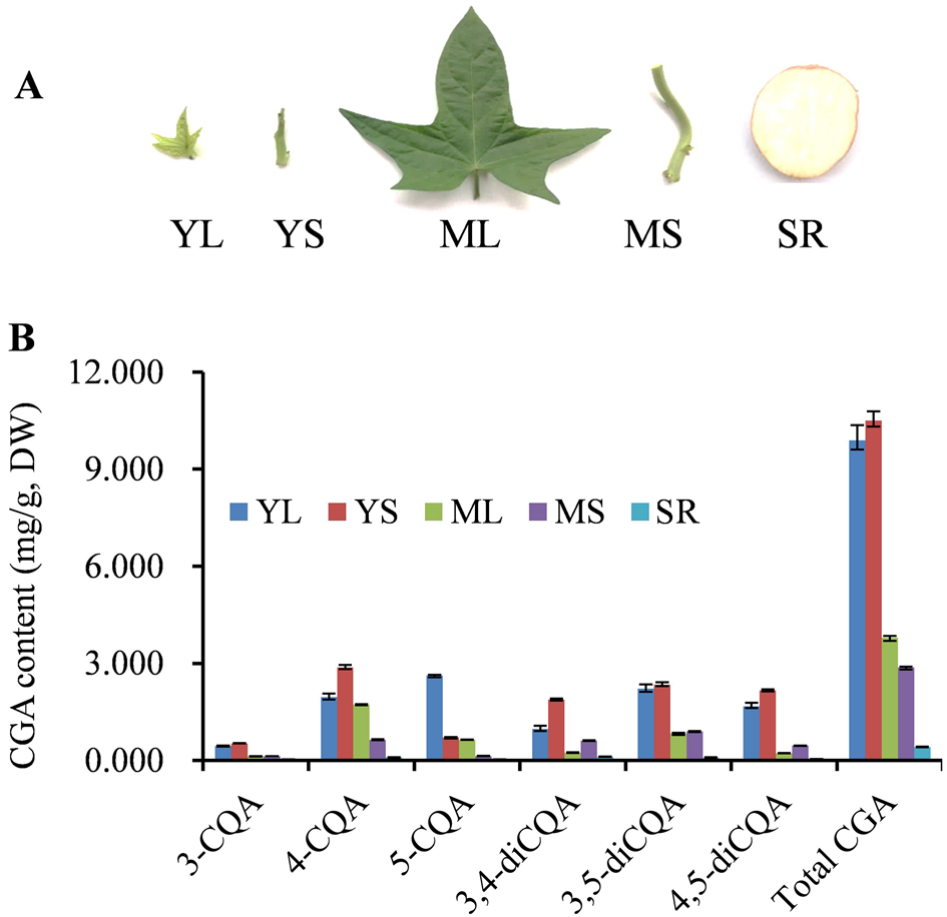
CGA content in different sweet potato tissues. (**A**) Young leaves (YL), mature leaves (ML), young stems (YS), mature stems (MS) and storage roots (SR) of sweet potato used for transcriptome sequencing and CGA content analysis. (**B**) Contents of 3-CQA, 4-CQA, 5-CQA, 3,4-diCQA, 3,5-diCQA, 4,5-diCQA and total CGA (the total sum of six CGAs) in different sweet potato tissues.

Six CGAs in different tissues and developmental stages were quantified and analyzed by HPLC to evaluate the dynamic accumulation of CGA in sweet potato (Fig. 1A). All the six CGAs were detected at distinct levels in different tissues and developmental stages (Fig. 1B). Concomitantly, 5-O-caffeoylquinicacid (5-CQA) accumulation was highest in YL with a maximum of ~ 2.62 mg/g dry weight, while the remaining CQAs, including 3-caeoylqunic acid (3-CQA), 4-caeoylquinicacid (4-CQA), 3, 4-di-O-caffeoylquinic acid (3, 4-diCQA), 3, 5-di-O-caffeoylquinic acid (3, 5-diCQA) and 4, 5-di-O-caffeoylquinic acid (4, 5-diCQA), were the most abundant in YS. Notably, the six CGAs were less abundant in the storage roots; only 0.11% ~ 0.70% of the CGAs were present in YL and YS.

### Transcriptome sequencing and assembly

Comparative transcriptome analysis of the YL, YS, ML, MS, and SR was performed in triplicates through RNA sequencing to understand the molecular mechanism of CGA biosynthesis in sweet potato and identify the associated genes. After eliminating the adapter and low-quality reads, a total of 99.92 Gb clean data was obtained from 15 sample, and each sample yielded up to 5.73 Gb of the data. The GC content of the 15 samples were 46.32∼48.25%, with an average of 46.99%, and the Q30 base percentage in each sample was more than 92.12%. The mapped ratio of each sample ranged from 73.45% to 77.24% (Supplementary Table S1). The assembly yielded 167,860 transcripts with an average length and an N50 value of 1,340 bp and 1,889 bp, respectively. After the processing by Trinity and TGICL software, 167,860 transcripts were further assembled into 59,287 unigenes, with an average length of 1,067 bp and an N50 value of 1,720 bp (Table 1).

**Table 1:** Summary of sequence assembly.

Length range	Transcript	Unigene
300–500	37,103(22.10%)	23,288(39.28%)
500–1000	44,706(26.63%)	15,604(26.32%)
1000–2000	50,920(30.33%)	11,578(19.53%)
2000 +	35,131(20.93%)	8,817(14.87%)
Total Number	167,860	59,287
Total Length	225,075,207	63,303,583
N50 Length	1889	1720
Mean Length	1340.85	1067.75

### Functional annotation and KEGG characterization

A total of 40,781 unigenes (68.78%) were annotated by searching against public databases. Further, the unigenes matched to NR, COG, GO, KEGG, KOG, Pfam, Swissprot and eggnog databases were 39,784 (97.56%), 13,573 (33.28%), 25,126 (61.61%), 15,175 (37.21%), 22,349 (54.80%), 28,306 (69.41%), 25,625(62.84%) and 37,222 (91.27%), respectively (Supplementary Table S2). The KEGG pathway-based analysis helps understand the biological functions of genes. Accordingly, 14,930 unigenes (25.18%) were mapped to 130 KEGG pathways, among which 3,578 genes were found to be associated with metabolic pathways. Moreover, 1,180 out of the 3,578 genes were mapped to 32 pathways involved in secondary biosynthesis and metabolism (Supplementary Table S3). The phenylpropanoid biosynthesis pathway (ko00940) was the largest group, containing 230 unigenes, followed by ascorbate and aldarate metabolism (ko00053, 86 unigenes) and phenylalanine metabolism (ko00360, 79 unigenes). CGA is a form of phenylpropanoid natural products in plants, and formed via the phenylpropanoid biosynthesis pathway. Thus, identifying and characterizing unigenes involved in phenylpropanoid biosynthesis pathways will help us better understand CGA biosynthesis in sweet potatoes.

### Identification of differentially expressed genes (DEGs)

**Figure 2:**
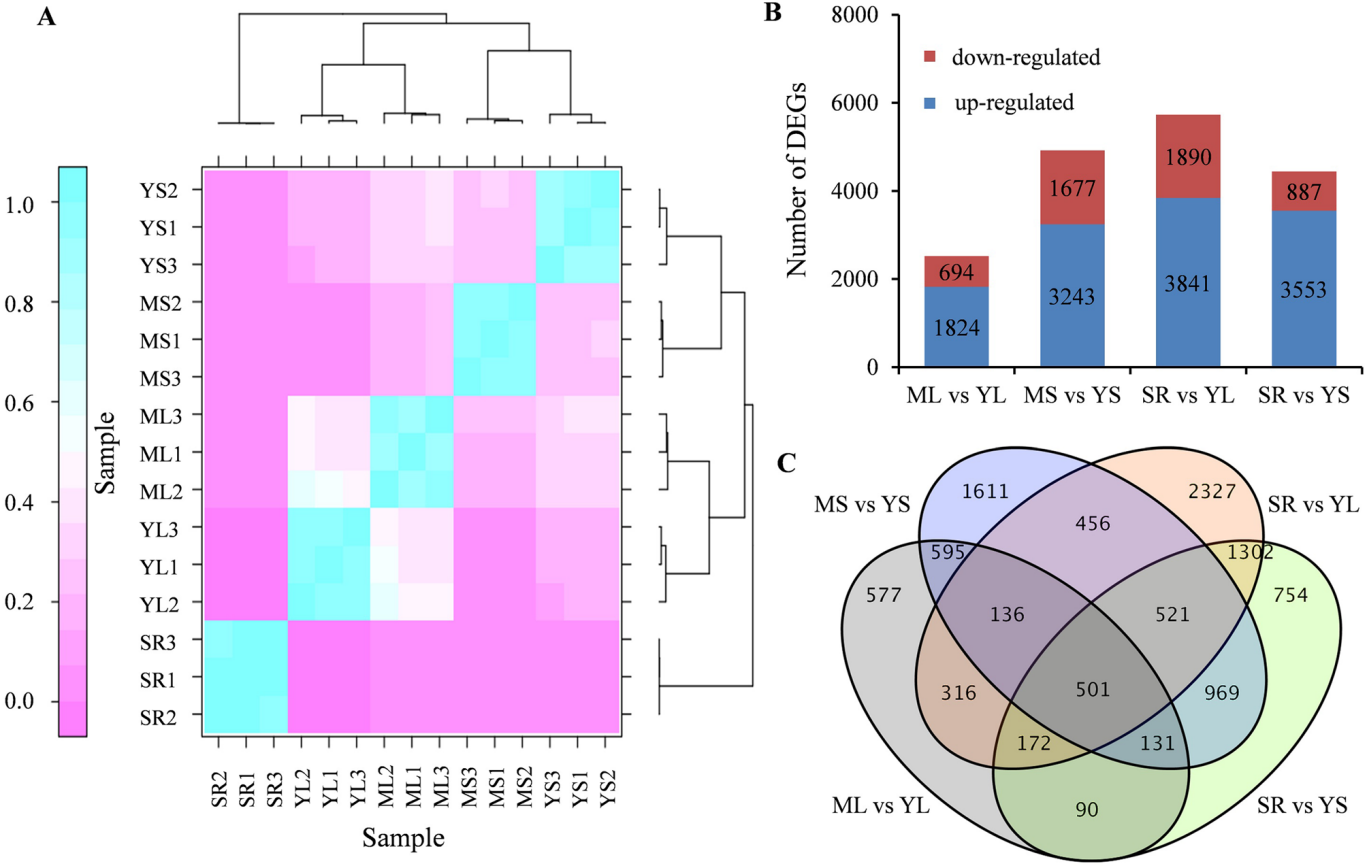
Correlation between samples and expression analysis of the differentially expressed genes. (**A**) The correlation heat map of samples. The left gradient barcode color indicates the minimum value (0) in lavender and the maximum (1) in light blue. A value close to 1 indicates a high positive correlationis, while a value close to 0 means no correlation. The heat map was drawn with DEseq (v3.5.1, http://www.bioconductor.org/packages/3.8/bioc/html/DESeq.html). (**B**) The number of up- or down-regulated DEGs in in different comparisons. (**C**) Venn diagram of the DEGs among the different groups. ML: mature leaves; YL: MS young: mature stems; leaves; YS: young stems; SR; storage roots.

The gene expression levels were evaluated using fragments per kilobase of exon per million fragments mapped (FPKM) values. Furthermore, correlation analysis revealed that the 15 samples could be divided into five groups and that the replicates had a strong positive correlation (r > 0.89), suggesting high reproducibility and reliability of the transcriptome data (Fig. 2A and Supplementary Table S4). The sample transcriptome data exhibiting noticeable differences in CGA accumulation were compared to identify candidate genes involved in CGA accumulation in sweet potato. In total, 10,458 DEGs were obtained by comparing the five samples in pairs. For the pairs, ML vs. YL, MS vs. YS, SR vs. YL and SR vs. YS, the differentially expressed unigenes were 2,518, 4920, 5,731 and 4,440, respectively (Fig. 2B). Specifically, the genes were up-regulated more than they were down-regulated. Additionally, 501 common unigenes were differentially expressed across the four comparison categories. The specific DEGs in ML vs. YL, MS vs. YS, SR vs. YL, and SR vs. YS were 577, 1,611, 2,307, and 754, respectively (Fig. 2C, Supplementary Table S5 and Supplementary Fig. S1).

Functional GO and KEGG enrichment analyses were performed to evaluate the functional categories of the 501 common DEGs. Functional GO analysis showed that 314 common DEGs were categorized into 31 functional groups, and these were further divided into three categories: biological process, cellular component and molecular function (Supplementary Fig. S2 and Supplementary Table S6). For the biological processes classification, the most abundant groups were metabolic processes (181), cellular processes (147), and single-organism processes (136). The most abundant groups within the molecular function category were catalytic activity (197) and binding (121). Finally, the highest categories were membrane (138), followed by cell (155) and cell part (155) in the cellular components category. Meanwhile, KEGG enrichment results showed that common DEGs were mainly enriched in phenylpropanoid biosynthesis, flavonoid biosynthesis and phenylalanine metabolism (Supplementary Fig. S3 and Supplementary Table S7), indicating that these metabolic pathways may be highly correlated to CGA accumulation in sweet potatoes.

### Candidate genes involved in the chlorogenic acid biosynthesis pathway

**Figure 3:**
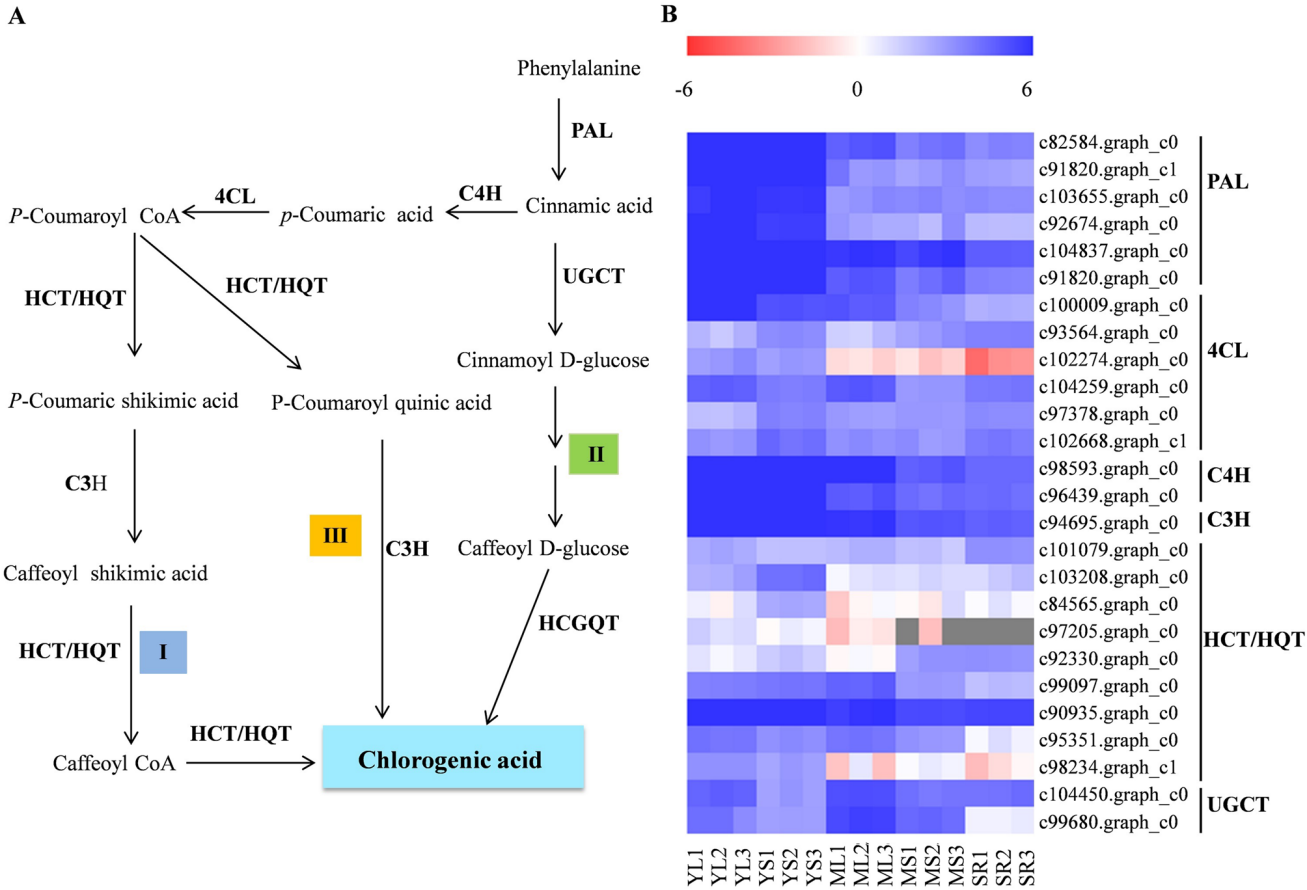
Expression of CGA biosynthesis pathway structural genes in sweet potato. (**A**) The three proposed CGA biosynthesis routes in sweet potato marked I, II and III. The genes involved in CGA biosynthesis including L-phenylalanine ammo-nialyase (PAL), 4-coumarate:CoA ligase (4CL), cinnamate 4-hydroxylase (C4H), P-coumarate 3-hydroxylase (C3H), Shikimate O-hydroxycinnamoyltransferase (HCT/HQT), UDP-glucose: cinnamate glucosyltransferase (UGCT) and hydroxyl cinnamoyl D-glucose:quinate hydroxycinnamoyl transferase (HCGQT). (**B**) Analysis of CGA-associated DEGs in different sweet potato tissues. The expression pattern of each gene is shown in heat map based on Log2 of FPKM values. Heat maps were generated using the MultiExperiment Viewer software (MeV v4.9.0, http://www.tm4.org/).

CGA biosynthesis has been proposed to occur via three alternative routes (Fig. 3A). A total of 56 potential genes, including eight *PAL*, twenty-five *4CL*, three *C4H*, thirteen *HCT/HQT*, four *C3H*, and three *UGCT* encoding putative enzymes involved in the CGA biosynthesis, were identified based on transcriptome data (Table 2 and Supplementary Table S8). However, no gene encoding HCGQT in the second route was detected in transcriptome data. Meanwhile, differential expression analysis showed that 28 unigenes were identified as DEGs (Fig. 3B and Supplementary Table S8). Among the DEGs, six *PAL* (c82584.graph_c0, c91820.graph_c1 c103655.graph_c0, c92674.graph_c0, c104837.graph_c0 and c91820.graph_c0), two *4CL* (c100009.graph_c0 and c102274.graph_c0), two *C4H* (c98593.graph_c0 and c96439.graph_c0), one *C3H* (c94695.graph_c0) and two *HCT/HQT* (c90935.graph_c0, c95351.graph_c0) showed high expression levels in YL and YS, and relatively low expressed levels in SR. These findings were consistent with CGA accumulation (Fig. 3B), suggesting the probable implication of the 28 unigenes in the CGA biosynthesis. Interestingly, there was no correlation between the expression of *UGCT* genes and CGA accumulation.

**Table 2:** The numbers of unigene involved in CGA biosynthesis.

Gene	EC number	Numbers
L-phenylalanin ammo-nialyase (PAL)	EC:4.3.1.24	8
4-Coumarate:CoA ligase (4CL)	EC:6.2.1.12	25
Cinnamate 4-hydroxylase (C4H)	EC:1.14.13.11	3
Shikimate O-hydroxycinnamoyltransferase (HQT/HCT)	EC:2.3.1.133	13
P-coumarate 3-hydroxylase (C3H)	EC:1.14.13.36	4
UDP-glucose: cinnamate glucosyltransferase (UGCT)	EC 2.4.1.177	3
Total		56

### Molecular and expression characterization of HCT/HQT

**Figure 4:**
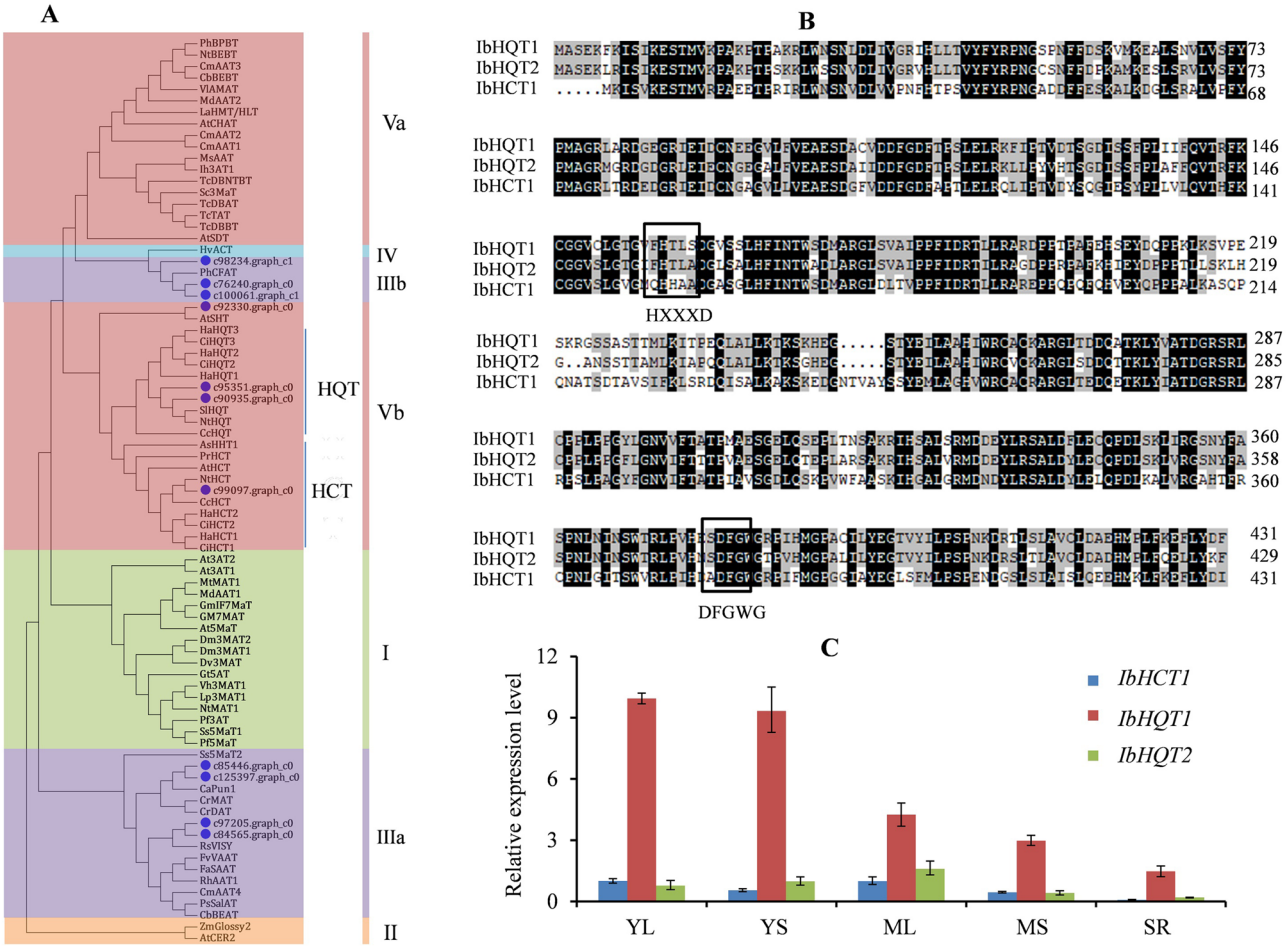
Characteristic and transcriptional expression of *IbHCT/HQTs*. (**A**) Phylogenetic analysis of IbHCT/HQTs and other BAHD proteins. The phylogenetic tree was established with neighbor-joining method by using program MEGA (Version 9.0, https://www.megasoftware.netExternal Link) with 1000 bootstrap replications. (**B**) Sequence alignment of IbHCT/HQTs. The amino acids in the box represent the conserved motifs HXXXD and DFGWG. The amino-acid sequences of BAHD proteins used in phylogenetic analysis and sequence alignment are listed in (Supplementary Table S9. Multiple alignments of amino acid sequences were performed using DNAMAN (version 8.0). (**C**) Relative expression levels of *IbHCT/HQTs* in different sweet potato tissues by qRT-PCR. The values are means ± SD of three independent biological replicates.

Considering the important roles of HCT/HQT in CGA biosynthesis, molecular characterization and expression of *HCT/HQ*T genes were further investigated. Three out of thirteen unigenes were predicted as complete full-length ORFs, and the other partial unigenes were further blasted against the genome databases to obtain full-length sequences. Phylogenetic analysis showed that all characterized BAHD proteins were divided into five distinct clades (I–V) and HCT/HQT proteins belonged to group V (Fig. 4A), which was similar to similar to results reported previously^8^. Four sequences (c85446.graph_c0, c125397.graph_c0, c97205.graph_c0 and c84565.graph_c0) belonged to clade IIIa and three sequences belonged to clade IIIb (c98234.graph_c1, c76240.graph_c0 and c100061.graph_c1), which were involved in the modification of alkaloid compounds and volatile ester biosynthesis^8^. Sequences of c90935.graph_c0 (IbHQT1) and c95351.graph_c0 (IbHQT2) clustered together with HQTs in clade Vb, while c99097.graph_c0 (IbHCT1) assembled with HCTs in clade Vb. Sequences of c99097.graph_c0 in clade Vb had a close evolutionary relationship with AtSHT in *Arabidopsis thaliana* that characterized as a spermidine hydroxycinnamoyl transferase^22^. Multiple alignments of the deduced amino acid sequences indicated that IbHQT1, IbHQT2 and IbHCT1 had typical structural characteristics of HCT/HQT proteins, exhibiting the conserved HXXXD and DFGWG motifs (Fig. 4B). Therefore, *IbHQT1*, *IbHQT2*, and *IbHCT1* are postulated to be the *HQT/HCT* genes in sweet potatoes.

To investigate the correlation between the CQA content and *HQT/HCT* expression levels, we measured the expression profiles of *IbHQT1*, *IbHQT2* and *IbHCT1* using qRT-PCR. The results indicated that the *IbHQT1* transcript levels in different tissues and developmental stages were significantly higher than those of *IbHQT2* and *IbHCT1* (Fig. 4C), and showed a significant correlation with CQA content, probably enhancing CQA biosynthesis.

### Candidate transcription factors involved in chlorogenic acid biosynthesis

**Figure 5:**
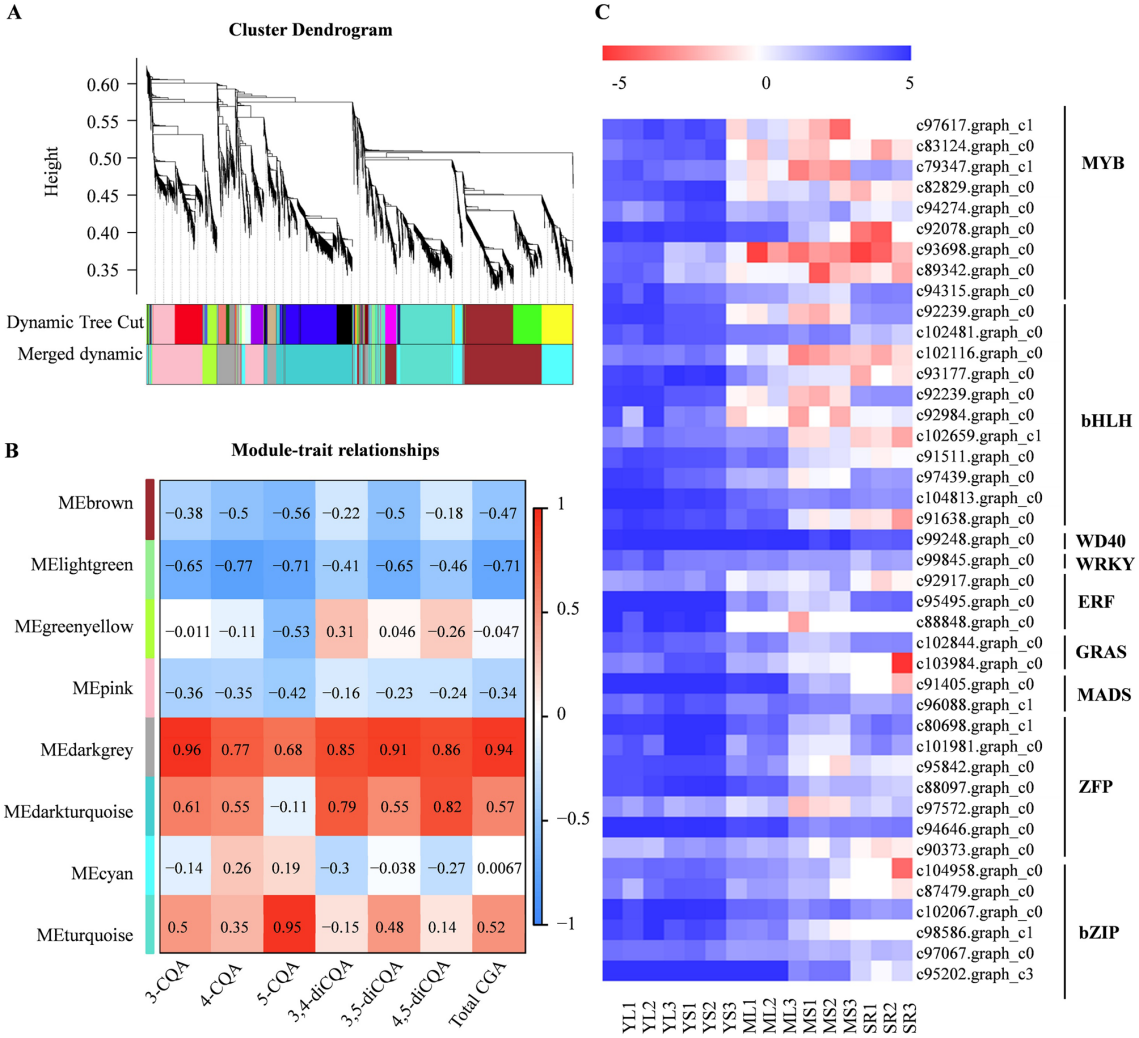
Gene co-expression networks associated with CGA metabolites generated by WGCNA. (**A**) Dendrogram showing modules identified by the weighted gene co-expression network analysis (WGCNA) and expressed genes clustering dendrogram. (**B**) Module-CGA weight correlations and corresponding *P*-values. The left panel shows the eight modules, while the right panel shows positive (red, 1) and negative (blue, − 1) correlations. Values close to –1 or 1 indicate a strong positive or negative linear relationship, while values close to 0 indicate weak correlation. (C) Heat map for the expression of transcription factor genes in the darkgrey module based on Log2 of FPKM values. Heat maps were generated using the MultiExperiment Viewer software (MeV v4.9.0, http://www.tm4.org/).

Plant phenylpropanoid metabolism is often regulated by transcription factors targeting the structural genes encoding enzymes. A gene co-expression network was constructed by WGCNA and compared with CQA contents in different tissues and developmental stages to screen the candidate transcription factors regulating CQA accumulation in sweet potato. In total, 9,408 DEGs were divided into eight distinct co-expression modules based on the similarity of expression profiles (Fig. 5A). Among the modules, the turquoise module was found to be highly positively related to 5-CQA accumulation (0.95); while the correlation coefficient of the darkgrey module and that of with the other CGA components, such as 3-CQA, 4-CQA, 3,4-diCQA, 3,5-diCQA, 4,5-diCQA, and total CQA was highest, which were 0.96, 0.77, 0.85, 0.91, 0.86 and 0.94, respectively (Fig. 5B). This finding suggested that the darkgrey module was highly correlated with the CGA accumulation in sweet potatoes.

Further analysis showed that darkgrey module contains 12 genes required for the CQA biosynthesis pathway. The genes included six *PALs* (c82584.graph_c0, c91820.graph_c1 c103655.graph_c0, c92674.graph_c0, c104837.graph_c0 and c91820.graph_c0), one *4CL* (c102274.graph_c0), two *C4Hs* (c98593.graph_c0 and c96439.graph_c0), one *C3H* (c94695.graph_c0) and one *HCT/HQT* (c90935.graph_c0), further substantiating the positive correlation between the darkgrey module and CQA biosynthesis. Among the 730 genes of darkgrey module, 42 were regulatory transcription factors, including nine *MYBs*, eleven *bHLHs*, seven zinc finger proteins (*ZFPs*), six *bZIPs*, three *ERFs*, two *GARSs*, two *MADSs*, one *WRKY* and one *WD40* (Supplementary Table S10). These transcription factors showed similar expression patterns with those of the genes encoding CQA biosynthesis enzymes (Fig. 5C), suggesting they may participate in regulating CGA biosynthesis in sweet potato.

### Validation of DEGs via qRT‑PCR Analysis

**Figure 6:**
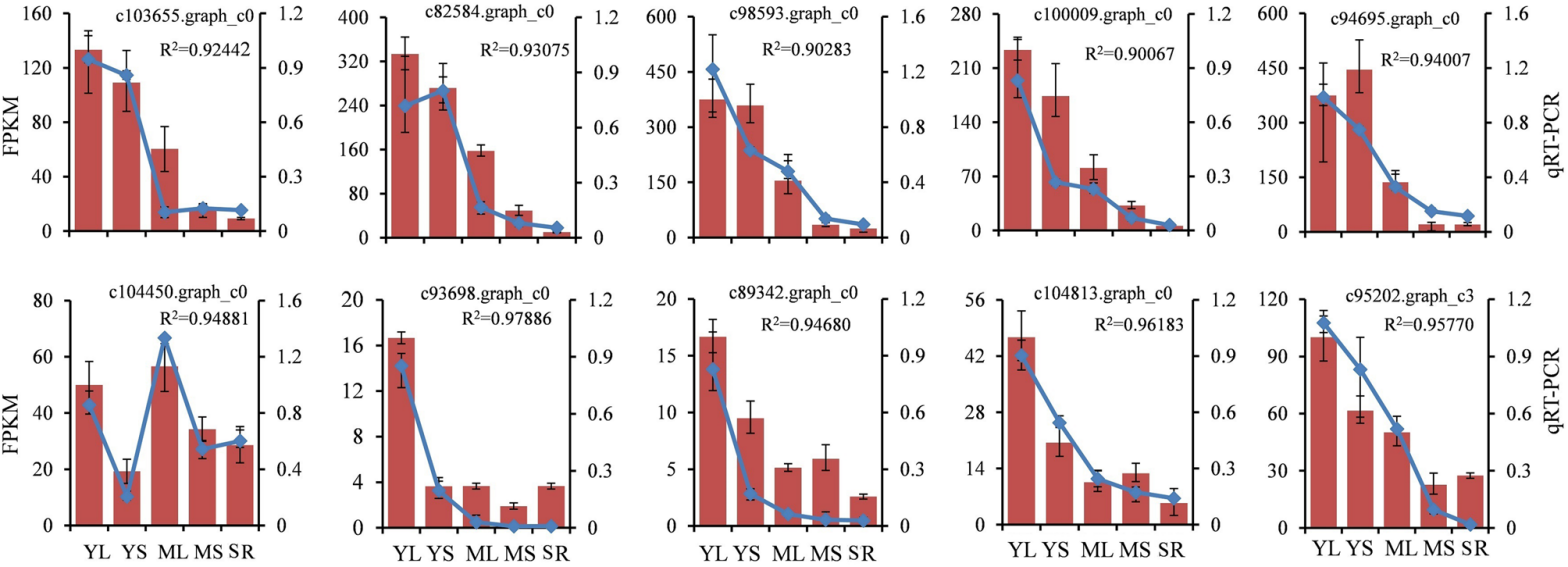
Expression patterns of the selected differentially expressed genes validated by qRT-PCR analysis. The line charts show the 10 unigenes qRT-PCR results involved in CGA biosynthesis and regulation in young leaves (YL), mature leaves (ML), young stems (YS), mature stems (MS), and storage roots (SR) of sweet potato; the histograms show the expression levels of these unigenes based on FPKM values. The values are means ± SD of three independent biological replicates. R^2^ represents correlation analysis between qRT-PCR and RNA-Seq data (FPKM values).

Ten DEGs associated with CGA biosynthesis were chosen for qRT-PCR assay to validate the reliability of gene expression data obtained from RNA-seq. As a result, all the 10 genes exhibited a similar pattern as those displayed by the FPKM values (R^2^ > 0.9) (Fig. 6). Therefore, the RNA-Seq results might provide reliable data for further research on CGA biosynthesis and its regulation in sweet potato.

## Discussion

Chlorogenic acids (CGAs) are widely distributed in sweet potato, contributing to biotic and abiotic stress resistance and nutritional benefits^18,20,23^. To date, most studies on sweet potato CGA mainly focus on extraction methods, and functional activities. However, very little is known about CGA biosynthesis mechanisms and their regulation in sweet potatoes^21^. Although CGAs have been reported in sweet potatoes, previous studies have not quantified and characterized CGA content in different tissues and different developmental stages. In this study, CGAs were found to be most abundant in young leaves and young stems, whereas much lower levels were detected in storage roots. Therefore, this study attempted to determine the potential genes responsible for CGA biosynthesis through comparative transcriptome combined with CGA content analyses in different tissues and developmental stages.

Three possible routes have been reported for the CGA biosynthesis (Fig. 2A). Putative genes encoding the key enzymes associated with the first and third routes of CGA biosynthesis were identified in the assembly. Phenylalanine ammonia-lyase (PAL), the first rate-limiting enzyme of the phenylpropanoid metabolism, has been demonstrated to play a crucial role in CGA synthesis^24–27^. Furthermore, we found that all the six differentially expressed *PAL* genes exhibited similar expression patterns that positively correlated changes in with CGA levels, suggesting an essential role of PAL in CGA accumulation. Recently, it has been confirmed that *IbPAL1* (c82584.graph_c0) overexpression in sweet potato significantly enhanced CGA accumulation^20^. C4H and 4CL (the early enzymes of phenylpropanoid metabolism) in CGA biosynthesis are yet to be understood^27^. However, our findings showed that two *C4H* genes (c98593.graph_c0 and c96439.graph_c0) and one *4CL* (c102274.graph_c0) exhibited a similar expression pattern with CGA concentrations, implying that they may be involved in CGA biosynthesis. The function of C3H in CGA biosynthesis has been determined by enzyme assays and gene overexpression in various plants^28–30^. Similarly, this study, expression of a *C3H* gene (c94695.graph_c0) was correlated with CQA content, which may be involved in CGA biosynthesis. Additionally, HQT/HCTs have been shown to play vital roles in CGA biosynthesis^9,31^. In the present study, three out of 13 potential HQT/HCT sequences (HbHQT1, HbHQT2 and HbHCT1) presented HXXXD and DFGWG conserved motifs, and were identified as HCT/HQT proteins. Among the three *HCT/HQT* genes, *HbHQT1* exhibited relatively high abundance in different tissues and developmental stages than *HbHQT2* and *HbHCT1*. Moreover, *HbHQT1* expression significantly correlated with CQA content, suggesting its implication in CGA biosynthesis. Although the three genes encoding UDP-glucose: cinnamate glucosyltransferase (UGCT) in the second route of CGA biosynthesis were identified, their expression did not correlate with CGA levels in different tissues and developmental stages of sweet potato. HCGQT, catalyzing caffeoyl-D-glucose and quinic acid to form CGA, was postulated to be the key enzyme of the second CGA biosynthesis pathway^6^. However, no corresponding *HCGQT* gene has been identified in plants to date. In addition, chlorogenic acid: glucaric acid caffeoyltransferase (CQT) in tomato was shown to catalyze the transfer of caffeic acid from CGA to glucaric and galactaric acids, indicating a possible CGA recycling route in plants^32^. Thus, the first and third routes may be the main CGA biosynthesis pathways in sweet potato. Nonetheless, more research is needed to understand the significance of the second CGA biosynthesis route.

Definite roles of the transcription factors in phenylpropanoid biosynthetic pathway regulation have been well established; however, less is known about the transcription factors regulating CGA biosynthesis^10,33^. Previous works have shown that the transcription factors involved in flavonoids biosynthesis might also regulate CGA biosynthesis^34,35^. Following the DEG and WGCNA analyses, we identified 42 transcription factor genes from nine families (MYB, bHLH, WD40, WRKY, ERF, GRAS, MADS, ZFP and bZIP) in the darkgrey module that might be involved in CGA biosynthesis (Supplementary Table S10 and Fig. 5C). These genes had a similar expression pattern across the different tissues and developmental stages and were co-expressed with CGA biosynthesis-related structural genes. Among the genes, c89342.graph_c0 was homologous to the Arabidopsis *AtPAP1/AtMYB75* gene encoding an MYB transcription factor implicated in anthocyanin biosynthesis. In addition, overexpression of *AtPAP1/AtMYB75* in *Platycodon grandiflorum* and *Leonurus Sibiricuscan* increased CGA content^34,35^. Conversely, c93698.graph_c0 showed high similarity to the Arabidopsis AtMYB12, which was identified as a flavonol-specific transcriptional activator in Arabidopsis. Arabidopsis gene *AtMYB12* also regulated CGA biosynthesis in tomato^36–38^. Moreover, *bHLH* (c104813.graph_c0, encoding GLABRA 3) and *bZIP* (c95202.graph_c3, encoding HY5) genes were detected by darkgrey module, their homologous were considered to be key transcription factors controlling anthocyanin biosynthesis^39,40^. In addition, LjbZIP8 could act as a transcriptional repressor in regulating *PAL2* expression and CGA content in *Lonicera japonica*^41^. Recent studies have revealed that the WRKY transcription factors acting as *HCT2* activators in poplar might also play an important role in CGA biosynthesis^10^. These studies showed the importance of transcription factors in regulating CGA biosynthesis. Although no reports on CGA biosynthesis regulation by WD40, ERF, GRAS, MADS and ZFP transcription factors have been published so far, these transcription factors have been reported to regulate other phenylpropanoids such as flavonoids and lignins^42–48^, suggesting their possible participation in CGA biosynthesis regulation. Therefore, understanding the specific functions of the 42 transcription factors in regulating sweet potato CGA biosynthesis need further studies.

In summary, comparative transcriptome and CGA changes in different sweet potato tissues and developmental stages were systematically investigated. The CGA accumulation varied among the tissues and developmental stages, and was abundant in YL and YS. Moreover, 59,287 unigenes were obtained, 3,767 of which were involved in secondary metabolism pathways. A darkgrey module associated with CGA accumulation was identified by differential expression analysis and WGCNA. In this module, 12 unigenes encoding crucial enzymes (PAL, 4CL, C4H, C3H and HCT/HQT) and 42 unigenes encoding transcription factors (MYB, bHLH, WD40, WRKY, ERF, MADS, GARS, bZIP and zinc finger protein) in darkgrey module may play essential roles in CGA biosynthesis. These results provide a basis for future research on the biosynthesis and regulation of the CGA metabolism pathway in sweet potatoes.

## Methods

### Plant materials

Sweet potatoes (QS80-12–11) used in this study were grown on a plantation in Hainan Academy of Agricultural Sciences, Hainan, China. The samples from different tissues and developmental stages (young leaves, mature leaves, young stems, mature stems, and storage roots) were collected at 90 days after planting for RNA extraction and CGA content analysis (Fig. 1A). Each sample was collected from at least three individual plants, and all experiments were conducted in triplicate. A portion of the samples was immediately frozen in liquid nitrogen, and stored at -80 °C until RNA extraction. The other samples portion was dried in a blast drier (ZHICHENG, Shanghai, China) at 60 °C and ground for CGA measurement analysis.

#### Estimation of chlorogenic acid content

The ground samples (0.2 g) were mixed with 1.5 ml of ethanol (70%, v/v) and then incubated in a water bath at 60 °C for 1 h. The mixture was then centrifuged at 5,000 g rpm for 10 min. Thereafter, the supernatant liquid was blown dry using nitrogen, re-dissolved in 1 ml methanol, and then filtered through 0.22 μm membrane filter for CGA analysis. Additionally, HPLC analysis was performed using an Ultimate 3000 system (Dionex, Sunnyvale, CA, USA) with a Waters Acuquity HSS T3 column (2.1 × 100 mm,1.8 μm) and a mixture of solvent A (0.1% formic acid in acetonitrile) and solvent B (0.1% aqueous solution of formic acid) as the mobile phase. The column oven temperature was maintained at 40 °C, and the flow rate was set at 0.3 ml/min. Typically, the linear gradient elution was programmed as follows: 10% A from 0 to 2 min; 10% to 60% A from 2 to 10 min; holding at 60% A from 10 to 15 min; 60% to 10% A from 15 to 15.1 min; holding at 10% A from 15.1 to 20 min. The eluting compounds were detected by monitoring at 326 nm. Subsequently, the CGA compounds were identified by comparing with the standard reagents, including 3-caeoylqunic acid (3-CQA), 4-caeoylquinicacid (4-CQA), 5-O-caffeoylquinicacid (5-CQA), 3, 4-di-O-caffeoylquinic acid (3, 4-diCQA), 3, 5-di-O-caffeoylquinic acid (3, 5-diCQA) and 4, 5-di-O-caffeoylquinic acid (4, 5-diCQA) (Sigma, St. Louis, MO, USA).

### RNA-Seq and gene co-expression analysis

Total RNA was extracted using RNAprep pure Plant Kit (Tiangen, Beijing, China) and subsequently used for cDNA library construction according to the Illumina manufacturer’s instructions. Furthermore, transcriptome sequencing was performed on the Illumina HiSeqTM 4000 platform at Biomarker Technologies Corporation (Beijing, China). Clean reads were obtained by eliminating the reads containing sequencing adapters, poly-N, and low-quality reads using SeqPrep (https://github.com/jstjohn/SeqPrep). De novo assembly of high-quality clean reads was performed using Trinity v2.5.1 (http://trinityrnaseq.sourceforge.net/)^49^, and then further processed with TGICL software to generate unigenes^50^. The BLAST software (http://www.ncbi.nlm.nih.gov/BLAST/) was employed for functional annotation of the unigenes against eight databases. These databases included NCBI non-redundant protein (NR) (http://www.ncbi.nlm.nih.gov)^51^, Gene Ontology (GO) (http://www.geneontology.org/)^52^, euKaryotic Orthologous Groups (KOG) (http://www.ncbi.nlm.nih.gov/COG/new/shokog.cgi)^53^, eggnog (http://eggnog5.embl.de/#/app/home)^54^, Protein family (Pfam)( http://www.sanger.ac.uk/Software/Pfam/)^55^, Swiss-Prot protein (http://www.expasy.ch/sprot)^56^, Kyoto Encyclopedia of Genes and Genomes (KEGG) (http://www.genome.jp/kegg)^57^and Clusters of Orthologous Groups of proteins (COG) (http://www.ncbi.nlm.nih.gov/COG)^58^. In addition, the gene expression level was calculated and normalized to fragments per kilobases of transcript per million fragments mapped (FPKM) values. Differentially expressed genes (DEGs) from different samples were identified by the DESeq2 software (version 1.12.4) based on the criteria of log2 |Fold Change|> 2, FDR < 0.001 and FPKM values > 2. Nonetheless, co-expression analysis was performed using the “Weighted Correlation Network Analysis (WGCNA)” package in R^59^. The data used in WGCNA analysis were components of the six CGA types and total CGA (sum of the six CGA components) compared with all RNA-seq genes.

### Bioinformatics analysis of HCT/HQT proteins

The open reading frames (ORFs) of *HQT/HCT* genes were predicted using the ORF finder (http://www.ncbi.nlm.nih.gov/), and full length of *HCT/HQT* unigenes with incomplete ORFs were obtained by scanning the unigenes against the sweet potato genome databases. All candidate HCT/HQT sequences were confirmed by Pfam (http://pfam.sanger.ac.uk/) and NCBI conserved domains database (CDD) (http://www.ncbi.nlm.nih.gov/cdd) databases. Eventually, a phylogenetic tree was established by MEGA 9.0 and Clustal X2.0 based on the candidate HCT/HQT protein sequences from sweet potato and other plants using the Neighbor-Joining method with 1000 bootstrap replicates.

### qRT‑PCR analysis

Quantitative real-time PCR (qRT-PCR) was employed to validate the reliability of gene expression using the RNA-seq data. The assay was performed in a Real-Time System Thermocycler using FastFire qPCR PreMix (Tiangen, Beijing, China). The conditions of the qRT-PCR amplification were as follows: initial denaturation at 95 °C for 3 min, followed by 40 cycles for 10 s at 95 °C, 15 s at 58 °C and 20 s at 72 °C. All reactions were performed in triplicates with specific primers (Supplementary Table S11), and β-actin was used as the reference gene. The relative gene expression level was calculated using the delta-delta Ct (2^−ΔΔCT^) method^60^.

### Statement on plant guidelines

Collection of plant material complies with relevant institutional, national, and international guidelines and legislation.

## Supplementary Information

Supplementary Information 1.

Supplementary Information 2.

## References

[1] Payyavula RS (2015). Synthesis and regulation of chlorogenic acid in potato: Rerouting phenylpropanoid flux in *HQT*-silenced lines. Plant Biotechnol. J..

[2] Clifford MN, Jaganath B, Ludwig IA, Crozier A (2017). Chlorogenic acids and the acyl-quinic acids: Discovery, biosynthesis, bioavailability and bioactivity. Nat. Prod. Rep..

[3] Clifford MN, Kerimi A, Williamson G (2020). Bioavailability and metabolism of chlorogenic acids (acyl-quinic acids) in humans. Compr. Rev. Food. Sci. Food Saf..

[4] He L (2013). Transcriptome analysis of buds and leaves using 454 pyrosequencing to discover genes associated with the biosynthesis of active ingredients in *Lonicera japonica* Thunb. PLoS ONE.

[5] Niggeweg R, Michael AJ, Martin C (2004). Engineering plants with increased levels of the antioxidant chlorogenic acid. Nat. Biotechnol..

[6] Villegas RJA, Kojima M (1986). Purification and characterization of hydroxycinnamoyl D-glucose quinate hydroxycinnamoyl transferase in the root of sweet potato, Ipomoea batatas Lam. J. Biol. Chem..

[7] Hoffmann L, Maury S, Martz F, Geoffroy P, Legrand M (2003). Purification, cloning, and properties of an acyltransferase controlling shikimate and quinate ester intermediates in phenylpropanoid metabolism. J. Biol. Chem..

[8] Tuominen LK, Johnson VE, Tsai CJ (2011). Differential phylogenetic expansions in BAHD acyltransferases across five angiosperm taxa and evidence of divergent expression among *Populus paralogues*. BMC Genom..

[9] Cheevarungnapakul K, Khaksar G, Panpetch P, Boonjing P, Sirikantaramas S (2019). Identification and functional characterization of genes involved in the biosynthesis of caffeoylquinic acids in Sunflower (*Helianthus annuus* L.). Front. Plant Sci..

[10] Zhang J (2018). Genome-wide association studies and expression-based quantitative trait loci analyses reveal roles of *HCT2* in caffeoylquinic acid biosynthesis and its regulation by defense-responsive transcription factors in Populus. New Phytol..

[11] Moglia A (2014). Dual catalytic activity of hydroxycinnamoyl-coenzyme A quinate transferase from tomato allows it to moonlight in the synthesis of both mono- and dicaffeoylquinic acids. Plant Physiol..

[12] Moglia A (2016). Genome-wide identification of BAHD acyltransferases and *in vivo* characterization of HQT-like enzymes involved in caffeoylquinic acid synthesis in *Globe Artichoke*. Front. Plant Sci..

[13] Chen ZX, Liu GH, Liu YQ, Xian ZQ, Tang N (2016). Overexpression of the *LmHQT1* gene increases chlorogenic acid production in *Lonicera macranthoides* Hand-Mazz. Acta. Physiol. Plant.

[14] Mudau SP (2018). Metabolomics-guided investigations of unintended effects of the expression of the hydroxycinnamoyl quinate hydroxycinnamoyltransferase (hqt1) gene from Cynara cardunculus var scolymus in Nicotiana tabacum cell cultures. Plant Physiol. Biochem..

[15] Li Y (2019). Correlation of the temporal and spatial expression patterns of *HQT* with the biosynthesis and accumulation of chlorogenic acid in *Lonicera japonica* flowers. Hortic. Res..

[16] Sheikha AFEI, Ray RC (2017). Potential impacts of bioprocessing of sweet potato: review. Crit. Rev. Food Sci. Nutr..

[17] Mohanraj R, Sivasankar S (2014). Sweet potato (*Ipomoea batatas* [L.] Lam)–a valuable medicinal food: A review. J. Med. Food..

[18] Zheng W, Clifford MN (2008). Profiling the chlorogenic acids of sweet potato (*Ipomoea batatas*) from China. Food Chem..

[19] Tanaka M, Ishiguro K, Oki T, Okuno S (2017). Functional components in sweet potato and their genetic improvement. Breed. Sci..

[20] Liao Y, Zeng L, Rao S, Gu D, Yang Z (2020). Induced biosynthesis of chlorogenic acid in sweetpotato leaves confers the resistance against sweet potato weevil attack. J. Adv. Res..

[21] Yu Y (2021). Overexpression of *IbPAL1* promotes chlorogenic acid biosynthesis in sweet potato. Crop J..

[22] Grienenberger E (2009). A BAHD acyltransferase is expressed in the tapetum of Arabidopsis anthers and is involved in the synthesis of hydroxycinnamoyl spermidines. Plant J..

[23] Santana-Gálvez J, Cisneros-Zevallos L, Jacobo-Velázquez DA (2017). Chlorogenic acid: Recent advances on its dual role as a food additive and a nutraceutical against metabolic syndrome. Molecules.

[24] Bate NJ (1994). Quantitative relationship between phenylalanine ammonia-lyase levels and phenylpropanoid accumulation in transgenic tobacco identifies a rate-determining step in natural product synthesis. Proc. Natl. Acad. Sci. USA.

[25] Shadle GL (2003). Phenylpropanoid compounds and disease resistance in transgenic tobacco with altered expression of L-phenylalanine ammonia-lyase. Phytochemistry.

[26] Zhang X, Liu CJ (2015). Multifaceted regulations of gateway enzyme phenylalanine ammonia-lyase in the biosynthesis of phenylpropanoids. Mol. Plant.

[27] Masuo S, Kobayashi Y, Oinuma K, Takaya N (2016). Alternative fermentation pathway of cinnamic acid production via phenyllactic acid. Appl. Microbiol. Biotechnol..

[28] Chen Z (2015). Transcriptome analysis reveals the mechanism underlying the production of a high quantity of chlorogenic acid in young leaves of Lonicera macranthoides Hand.-Mazz. PLoS ONE.

[29] Kim YB (2013). Characterization of genes for a putative hydroxycinnamoyl-coenzyme A quinate transferase and p-coumarate 3'-hydroxylase and chlorogenic acid accumulation in tartary buckwheat. J. Agric. Food Chem..

[30] Knollenberg BJ, Liu J, Yu S, Lin H, Tian L (2018). Cloning and functional characterization of a p-coumaroyl quinate/shikimate 3'-hydroxylase from potato (*Solanum tuberosum*). Biochem. Biophys. Res. Commun..

[31] Legrand G (2016). Identification and characterization of five BAHD acyltransferases involved in hydroxycinnamoyl ester metabolism in Chicory. Front. Plant Sci..

[32] Strack D, Gross W (1990). Properties and activity changes of chlorogenic acid: Glucaric acid caffeoyl-transferase from tomato (*Lycopersicon esculentum*). Plant Physiol..

[33] Dong NQ, Lin HX (2021). Contribution of phenylpropanoid metabolism to plant development and plant-environment interactions. J. Integr. Plant Biol..

[34] Tuan PA (2014). Enhancement of chlorogenic acid production in hairy roots of *Platycodon grandiflorum* by over-expression of an *Arabidopsis thaliana* transcription factor *AtPAP1*. Int. J. Mol. Sci..

[35] Sitarek P (2018). Over-expression of AtPAP1 transcriptional factor enhances phenolic acid production in transgenic roots of Leonurus sibiricus L. and their biological activities. Mol. Biotechnol..

[36] Mehrtens F, Kranz H, Bednarek P, Weisshaar B (2005). The Arabidopsis transcription factor MYB12 is a flavonol-specific regulator of phenylpropanoid biosynthesis. Plant Physiol..

[37] Luo J (2008). AtMYB12 regulates caffeoyl quinic acid and flavonol synthesis in tomato: Expression in fruit results in very high levels of both types of polyphenol. Plant J..

[38] Pandey A (2015). *AtMYB12* expression in tomato leads to large scale differential modulation in transcriptome and flavonoid content in leaf and fruit tissues. Sci. Rep..

[39] Feyissa DN, Løvdal T, Olsen KM, Slimestad R, Lillo C (2009). The endogenous *GL3*, but not *EGL3*, gene is necessary for anthocyanin accumulation as induced by nitrogen depletion in *Arabidopsis* rosette stage leaves. Planta.

[40] Liu CC (2018). The bZip transcription factor HY5 mediates CRY1a-induced anthocyanin biosynthesis in tomato. Plant Cell Environ..

[41] Zha L (2017). DNA methylation influences chlorogenic acid biosynthesis in *Lonicera japonica* by mediating LjbZIP8 to regulate phenylalanine ammonia-lyase 2 expression. Front. Plant Sci..

[42] Habib S, Lwin YY, Li N (2021). Down-regulation of SlGRAS10 in tomato confers abiotic stress tolerance. Genes (Basel).

[43] Zhao C (2021). Three AP2/ERF family members modulate flavonoid synthesis by regulating type IV chalcone isomerase in citrus. Plant Biotechnol. J..

[44] Ma R (2017). AP2/ERF Transcription factor, Ii049, positively regulates lignan biosynthesis in *Isatis indigotica *through activating salicylic acid signaling and lignan/lignin pathway genes. Front. Plant Sci..

[45] Garceau DC, Batson MK, Pan IL (2017). Variations on a theme in fruit development: The PLE lineage of MADS-box genes in tomato (*TAGL1*) and other species. Planta.

[46] Tao Z (2017). BnLATE, a Cys2/His2-type zinc-finger protein, enhances silique shattering resistance by negatively regulating lignin accumulation in the silique walls of *Brassica napus*. PLoS ONE.

[47] Lian J (2017). Silencing of *BnTT1* family genes affects seed flavonoid biosynthesis and alters seed fatty acid composition in *Brassica napus*. Plant Sci..

[48] Tian H, Wang S (2020). TRANSPARENT TESTA GLABRA1, a Key Regulator in plants with multiple roles and multiple function mechanisms. Int. J. Mol. Sci..

[49] Grabherr MG (2011). Full-length transcriptome assembly from RNA-Seq data without a reference genome. Nat. Biotechnol..

[50] Pertea G (2003). TIGR Gene Indices clustering tools (TGICL): A software system for fast clustering of large EST datasets. Bioinformatics.

[51] Deng Y (2006). Integrated nr database in protein annotation system and its localization. Comput. Eng..

[52] Ashburner M (2000). Gene ontology: Tool for the unification of biology. The gene ontology consortium. Nat. Genet..

[53] Koonin EV (2004). A comprehensive evolutionary classification of proteins encoded in complete eukaryotic genomes. Genome Biol..

[54] Huerta-Cepas J (2016). eggNOG 45: A hierarchical orthology framework with improved functional annotations for eukaryotic, prokaryotic and viral sequences. Nucl. Acids Res..

[55] Finn RD (2014). Pfam: The protein families database. Nucleic Acids Res..

[56] Apweiler R (2004). UniProt: the Universal Protein knowledgebase. Nucl. Acids Res..

[57] Kanehisa M, Goto S, Kawashima S, Okuno Y, Hattori M (2004). The KEGG resource for deciphering the genome. Nucl. Acids Res..

[58] Tatusov RL (2003). The COG database: an updated version includes eukaryotes. BMC Bioinf..

[59] Langfelder P, Horvath S (2008). WGCNA: An R package for weighted correlation network analysis. BMC Bioinf..

[60] Schmittgen TD, Livak KJ (2008). Analyzing real-time PCR data by the comparative CT method. Nat. Methods.

