# Supplementary material for: Comparative transcriptome and weighted correlation network analyses reveal candidate genes involved in chlorogenic acid biosynthesis in sweet potato
Source: Sci Rep. 2022 Feb 17;12:2770. doi: 10.1038/s41598-022-06794-4 (PMC8854667; doi:10.1038/s41598-022-06794-4)
Supplement: Supplementary file 1 — Supplementary Information 1. [file 41598_2022_6794_MOESM1_ESM.docx]

# [Comparative transcriptome and weighted correlation network analyses reveal candidate genes involved in](http://eng.area.5read.com/views/specific/2929/FJourDetail.jsp?dxNumber=165470139456&d=9E2A62C81FAAD9FE76B34AFD4FA6E619&s=Comparative+transcriptome+analysis+reveals+genes+biosynthesis&fenlei=0) chlorogenic acid biosynthesis in sweet potato

Jing Xu ^1^, Jiahong Zhu ^2 ,^ *, Yanhui Lin ^1^, Honglin Zhu ^1^, Liqiong Tang ^1^, Benjun Hou ^1^, Xinhua Wang ^1^ & Xiaoning Wang ^1,^*

^1^Institute of Cereal Crops, Hainan Academy of Agricultural Sciences, Key Laboratory of Crop Genetics and Breeding of Hainan Province, Haikou, 571100, china

^2^Institute of Tropical Biosciences and Biotechnology, Chinese Academy of Tropical Agricultural Sciences, Haikou, 571101, china

Correspondence: [zhujiahong@itbb.org.cn](mailto:zhujiahong@itbb.org.cn) (J.H.Z.); [wxning2599@163.com](mailto:wxning2599@163.com) (X.N.W)


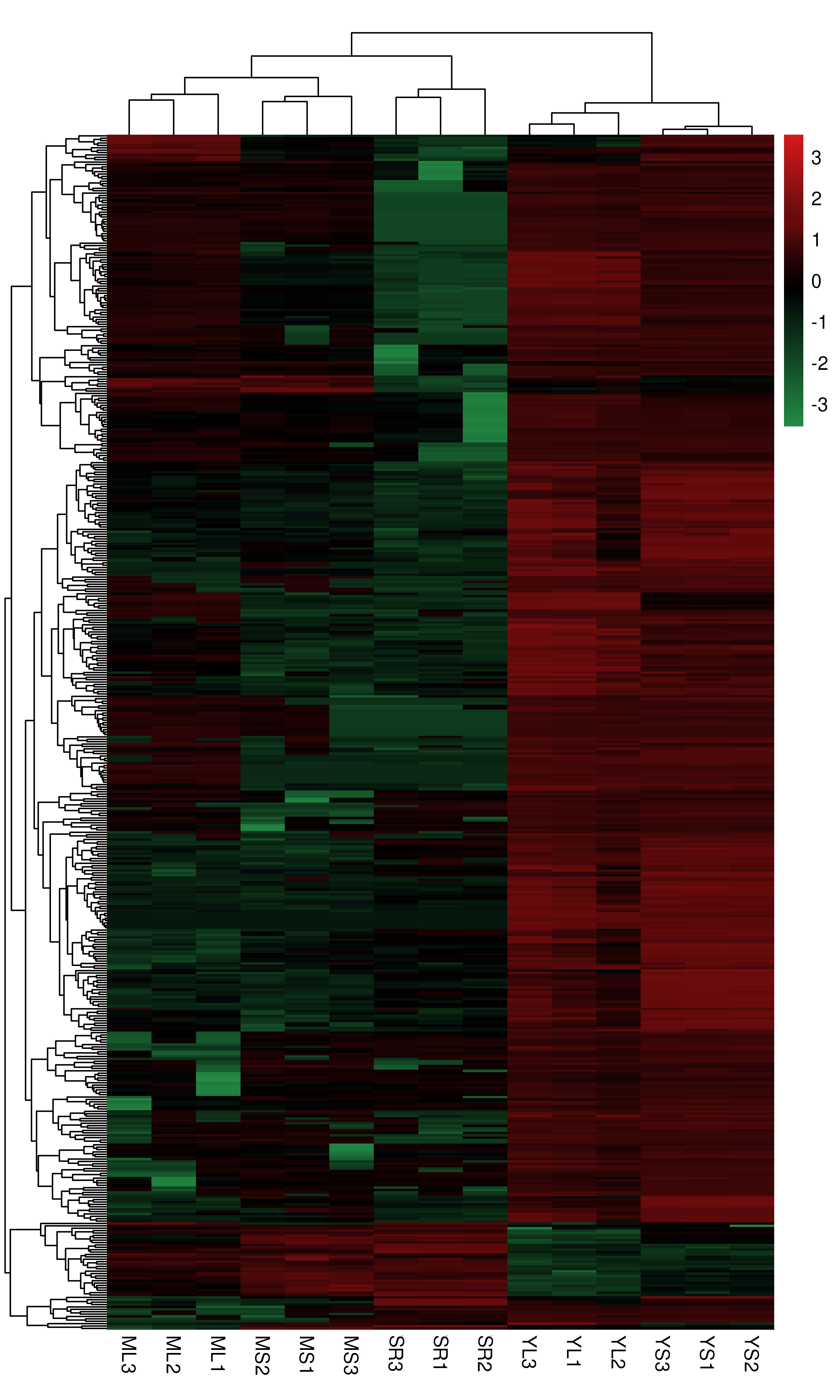


**Supplementary Figture S1.** The expression heatmap of common DEGs among different tissues.

Heat maps were generated using the MultiExperiment Viewer software (MeV v4.9.0, http://www.tm4.org/).


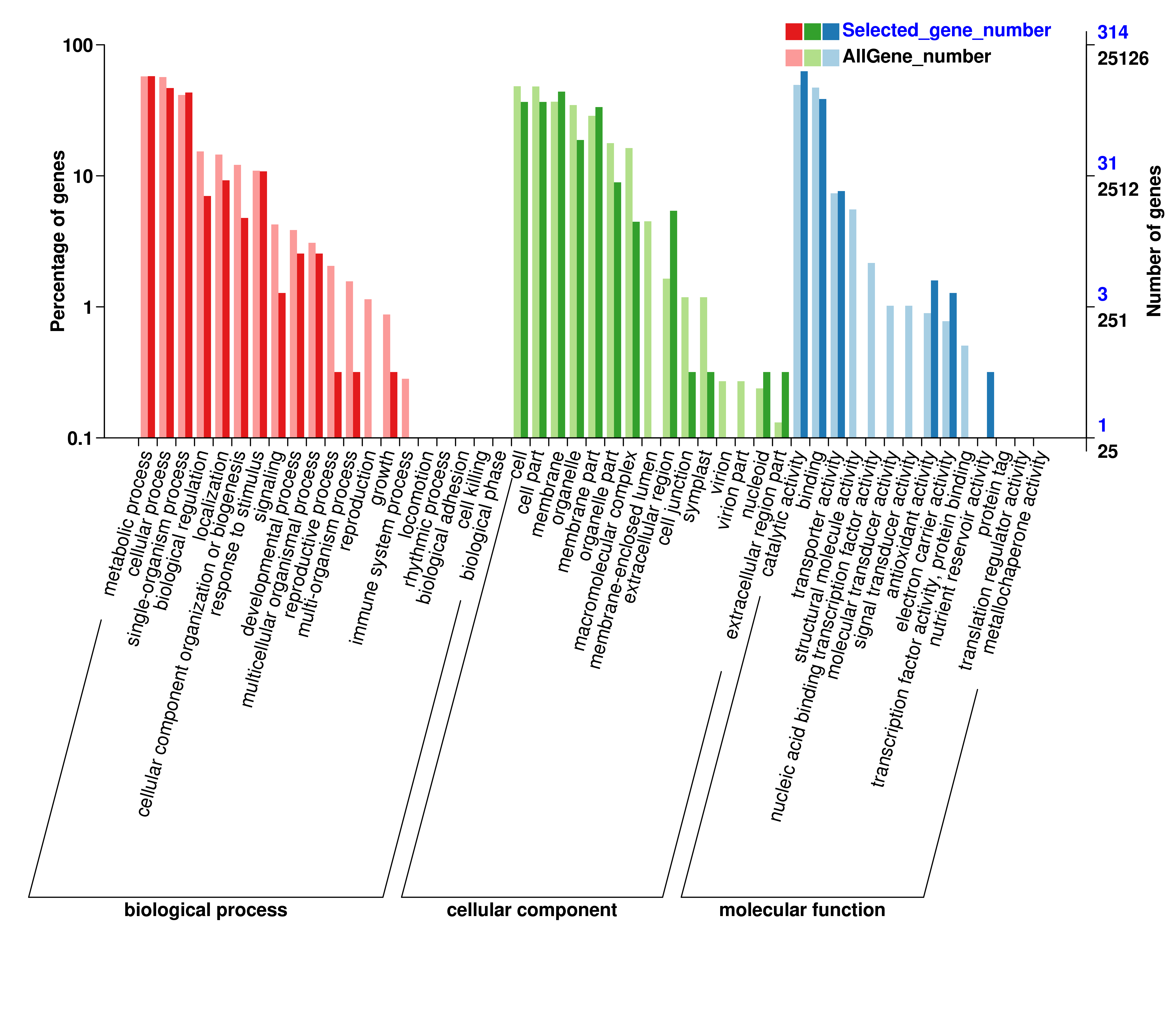


**Supplementary Figture S2.**  Functional GO analysis of common DEGs among different tissues. The figures was drawn with DEseq (v3.5.1). (<http://www.bioconductor.org/packages/3.8/bioc/html/DESeq.html>).


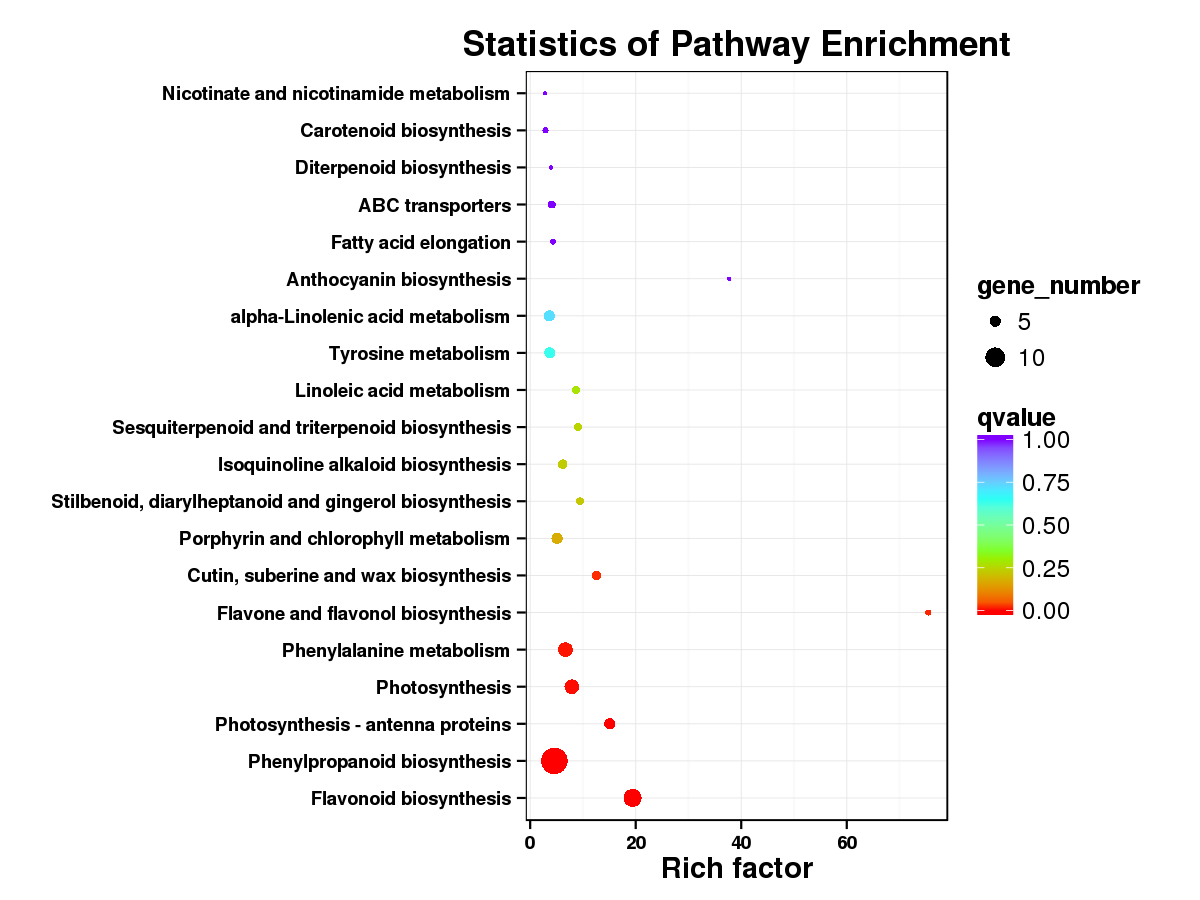


**Supplementary Figture S3.** KEGG enrichment analysis of common DEGs among different tissues. The figures was drawn with DEseq (v3.5.1). (<http://www.bioconductor.org/packages/3.8/bioc/html/DESeq.html>).
